# Should an Anesthesiologist Be Interested in the Patient’s Personality? Relationship Between Personality Traits and Preoperative Anesthesia Scales of Patients Enrolled for a Hip Replacement Surgery

**DOI:** 10.3390/jcm14155227

**Published:** 2025-07-24

**Authors:** Jakub Grabowski, Agnieszka Maryniak, Dariusz Kosson, Marcin Kolacz

**Affiliations:** 1 Faculty of Medicine, Medical University of Warsaw, 02-091 Warsaw, Poland; kubusg2000@gmail.com; 2 Faculty of Psychology, University of Warsaw, 00-183 Warsaw, Poland; agnieszka.maryniak@psych.uw.edu.pl; 3 Department of Anesthesiology and Intensive Care Education, Medical University of Warsaw, 02-005 Warsaw, Poland; dariusz.kosson@wum.edu.pl; 4 1st Department of Anesthesiology and Intensive Therapy, Medical University of Warsaw, 02-005 Warsaw, Poland

**Keywords:** psychological prophylaxis, operation, personality, anxiety

## Abstract

**Background:** Preparing patients for surgery considers assessing the patient’s somatic health, for example by the American Society of Anesthesiology (ASA) scale or the Revised Cardiac Risk Index (RCRI), known as the Lee index. This process usually ignores mental functioning (personality and anxiety), which is known to influence health. The purpose of this study is to analyze the existence of a relationship between personality traits (the Big Five model and trait-anxiety) and anesthesia scales (ASA scale, Lee index) used for the preoperative evaluation of patients. **Methods:** The study group comprised 102 patients (59 women, 43 men) scheduled for hip replacement surgery. Patients completed two psychological questionnaires: the NEO-FFI (NEO Five Factors Inventory) and the X-2 STAI (State-Trait Anxiety Inventory) sheet. Next, the presence and possible strength of the relationship between personality traits and demographic and medical variables were analyzed using Spearman’s rho rank correlation coefficient. **Results:** Patients with a high severity of trait anxiety are classified higher on the ASA scale (rs = 0.359; *p* < 0.001). Neuroticism, defined according to the Big Five model, significantly correlates with scales of preoperative patient assessment: the ASA classification (rs = 0.264; *p* < 0.001) and the Lee index (rs = 0.202; *p* = 0.044). A hierarchical regression model was created to test the possibility of predicting ASA scores based on personality. It explained more than 34% of the variance and was a good fit to the data (*p* < 0.05). The controlled variables of age and gender accounted for more than 23% of the variance. Personality indicators (trait anxiety, neuroticism) additionally accounted for slightly more than 11% of the variance. Trait anxiety (Beta = 0.293) proved to be a better predictor than neuroticism (Beta = 0.054). **Conclusions:** These results indicate that inclusion of personality screening in the preoperative patient evaluation might help to introduce a more individualized approach to patients, which could result in better surgical outcomes.

## 1. Introduction

The modern approach to perioperative care is based on the aim of reducing the stress before and after surgery. Less stress helps to accelerate the patient’s return to full activity [1]. Preparing patients for surgery usually takes into account the patient’s somatic health without considering their mental functioning. In contrast, according to the WHO definition: “Health is a state of complete physical, mental and social well-being and not merely the absence of disease or infirmity” [2].

Mental disorders, including anxiety disorders, are becoming more common among people. In Poland, this was shown by the studies called EZOP I: Epidemiologia zaburzeń psychiatrycznych i dostępność psychiatrycznej opieki zdrowotnej (Epidemiology of Mental Disorders and Access to Mental Health Care) and EZOP II [3,4].

### 1.1. Personality and Anxiety

Personality is one of the most complex psychological concepts. A modern definition of personality created by Lawrence Pervin describes it as “the complex organization of cognitions, affects, and behaviors that gives direction and pattern (coherence) to the person’s life. Like the body, personality consists of both structures and processes and reflects both nature (genes) and nurture (experience). In addition, personality includes the effects of the past, including memories of the past, as well as constructions of the present and future” [5]. Currently, one of the dominant theoretical models of personality is Costa and McCrae’s five-factor theory of personality—the Big Five model) [6,7]. Authors suggest that a description of the whole personality can be limited to only five personality traits, i.e., the general disposition to specific reactions, behaviors, feelings, thoughts, and motives over a range of situations, which is relatively constant and specific to an individual [8].

In Costa and McCrae’s five-factor theory of personality, said traits are described as follows [9]:

1. Neuroticism—in its extremes reflects emotional adjustment and emotional instability, expresses susceptibility to experiencing negative emotions (for example, fear, confusion, guilt) and sensitivity to psychological stress;
2. Extraversion—characterizes the quality and quantity of social interactions, the ability to experience positive emotions, and the level of energy and activity;
3. Openness to experience—describes the tendency to seek new life experiences, tolerance of the unfamiliar and cognitive curiosity;
4. Agreeableness—relates to attitudes towards other people, which can be understood as a sense of trust in others or lack thereof, sensitivity or indifference to strangers, a preference for cooperation, or a desire to compete;
5. Conscientiousness—characterizes the degree of organization, perseverance, and motivation in pursuit of a goal.

Personality traits are variables that are unavailable to direct observation and mediate between stimulus and response. They are continuous in nature and have a normal distribution in the population. If they are characterized by extreme intensity, they can lead to susceptibility to psychosomatic diseases, behavioral disorders and can be associated with maladaptive tendencies in behavior [9].

Anxiety is an unpleasant emotional state involving the experience of feelings of threat, tension, and mental discomfort, the source of which a person is unable to pinpoint [10]. In his research, Raymond Cattell distinguished two factors describing anxiety. The first is state anxiety, which varies from situation to situation, accompanied by stimulation of the sympathetic branch of the autonomic nervous system and psychological distress. The second is trait anxiety, responsible for the occurrence of individual differences in the intensity of the response to stressors [11].

### 1.2. Preoperative Anxiety and Health

There is convincing evidence that personality traits affect health and life expectancy [12]. Such an influence occurs through three interrelated mechanisms [12]. Firstly, the very traits in question may be connected to disease triggers. Secondly, personality impacts an individual’s behavioral patterns, which may be health-promoting or the opposite. Thirdly, personality traits can influence the degree to which medical recommendations and adherence to treatment regimens (compliance) are implemented in daily life [12]. The personality traits described in the Big Five factors have been shown to be a significant predictor of health [13]. A metasynthesis by Strickhouser et al. revealed a high correlation with mental health, a medium one with behaviors that affect health, and a low correlation with physical health. Despite the low association with somatic health that has been observed, researchers emphasize the importance of this connection, as it can accumulate over time [13].

Specific psychological profile, however, can have a direct relationship with somatic diseases (for example adhesive capsulitis) [14,15].

### 1.3. Preoperative Patient Assessment

The standard procedure in comprehensive perioperative care is the ERAS (Enhanced Recovery After Surgery) protocol. Following it makes it possible to shorten the length of hospitalization, reduce the risk of perioperative complications, and influence a better outcome of therapy [1]. It also includes recommendations for anesthesia. For safe anesthesia, preoperative patient assessment is essential. The most popular method of classifying a patient’s general condition is the ASA scale developed by the American Society of Anesthesiology (Table 1) [16].

In addition, prior to non-cardiac surgery, the European Society of Cardiology (ESC) and the European Society of Anesthesiology (ESA) recommend assessing the risk of cardiac complications according to the Revised Cardiac Risk Index (RCRI), popularly known as the Lee index (Table 2) [17].

### 1.4. Research Questions and Hypotheses

The purpose of this study is to analyze the existence of a relationship between personality traits (the Big Five model) and both anxiety (trait anxiety) and anesthesia scales for the preoperative evaluation of patients enrolled for hip replacement surgery. According to the current state of knowledge, somatically ill patients differ from healthy individuals in the mental characteristics mentioned above and are more likely to express anxiety [18]. Unfortunately, the results of most studies are not directly translated into clinical practice. The introduction of personality screening would make it possible to introduce a more individualized approach to patients, which could result in better surgical outcomes.

The following hypotheses were formulated:

1. Anxiety understood as a trait correlates with patient classification on the ASA scale and the Lee index;
2. Personality traits according to the Big Five model correlate with scales used for preoperative patient assessment.

## 2. Materials and Methods

### 2.1. Sample Characteristics

The study included patients enrolled for total hip replacement surgery by the Department of Orthopedics and Locomotor Traumatology at the Medical University of Warsaw. The approval of the Bioethical Committee of the Medical University of Warsaw (AKBE/352/2023) and the consent of the heads of various medical entities had previously been obtained. All procedures in the study were performed in accordance with ethical standards of the Helsinki Declaration. The inclusion criteria were the patient’s consent to participate in the study and an anesthesiology questionnaire on file with the physician’s classification of the patient into the appropriate group on the ASA scale and the Lee index. The exclusion criteria were a condition that prevented the patient from completing the questionnaire and lack of consent to participate in the study.

The study included 102 adults: 59 women (57.8%) and 43 men (42.2%) between the ages of 19 and 90 (M = 66.95 with SD = 12.81). Ninety-five percent of the respondents were from 40 to 88 years old. Most of them had already undergone orthopedic surgery when answering the questions (*n* = 68; 66.7%), while the rest were expecting surgery the following day (*n* = 34; 33.3%).

### 2.2. Questionnaires

The following questionnaires were used:

1. The NEO Five Factors Inventory (NEO-FFI) questionnaire to measure the main characteristics of the Five Factor Theory of Personality, in the Polish adaptation [9]. It is a shortened version of the original personality inventory (NEO-PI-R) made by Costa and McCrae. While creating it the authors had taken care that the psychometric characteristics were of high quality [19]. As a result, the inventory is empirically verifiable and did not encounter significant opposition from the community of personality psychology experts to most of the postulates that were proposed [20]. The NEO Five Factors Inventory (NEO-FFI) questionnaire was used because the original personality inventory (NEO-PI-R) is too long and it would take too much time for patients to fill it in. The results of previous research confirm that the NEO-FFI is a reliable and accurate tool for measuring personality in the Big Five model [21];
2. A questionnaire for assessing the severity of anxiety understood as both a state and trait according to the STAI (State-Trait Anxiety Inventory) by Charles Spielberger. It is used to determine the level of trait anxiety, understood as a fixed internal disposition of the people examined. It also provides an opportunity to record changes in the severity of state anxiety understood as the subjective and consciously perceived feelings of apprehension and tension occurring in response to specific external stressors [22]. A Polish adaptation was used [23]. Only the trait anxiety sheets (sheet X-2) were analyzed in the study;
3. An anesthesiology questionnaire prepared by the University Clinical Center of the Medical University of Warsaw with a physician’s classification on the ASA scale and the Lee index.

### 2.3. Research Procedure

The study was conducted between 4 January 2024 and 9 April 2024. Each participant was informed of the confidential nature of the study and the subsequent anonymization of data and gave informed consent to participate in the study.

The patients completed the psychological questionnaires themselves: the NEO-FFI and the X-2 STAI sheet. Two of them filled out only the STAI questionnaire. Therefore, 102 respondents (59 women, 43 men) were included in the analyses involving trait anxiety (STAI), and 100 subjects (59 women, 41 men) were considered in the analysis of the Big Five model (NEO-FFI).

The material prepared for analysis did not include any sensitive patient data.

### 2.4. Analysis

The raw scores on the NEO-FFI and STAI questionnaires were studied in relation to age and gender norms. This was not possible for 11 patients, because they were over 80 years old, which is the age limit for which the norm tables were developed for both tests. When normalizing the raw scores of these participants, the tables for the oldest age groups were used. As a result of this procedure, the severity of personality traits according to the Big Five theory and trait anxiety on the STen scale were obtained. STen scale was used in order to simplify comparison across individuals by converting raw scores to a common scale. It is a ten points scale, with 5.5 as a midpoint. Scores 1–3 are low, 4–7 average, and 8–10 high. Age and gender were also specified for inclusion as control variables in the hierarchical regression model.

### 2.5. Statistical Analysis

Further statistical analyses were carried out using the 29.0.2 version of SPSS program. Due to the relatively high number of observations, the distribution of the variables studied was checked using the Kolmogorov–Smirnov test.

Next, the presence and possible strength of the relationship between personality and demographic and medical variables were analyzed. To achieve this Spearman’s rho rank correlation coefficient and the Mann–Whitney U test for two non-parametric independent groups were used. Linear regression analysis was performed to assess the feasibility of estimating ASA scale values based on the intensity of the patient’s personality traits.

## 3. Results

The results of the Kolmogorov–Smirnov test with Lilliefors’ significance correction for all the labeled, quantitative variables, i.e., the ASA classification, Lee index, age, neuroticism (N), extraversion (E), openness to experience (O), agreeableness (Ag), conscientiousness (C), and trait anxiety (An), are statistically significant. This means that their distributions deviate from normal. Therefore, non-parametric tests were used in further analyses (Table 3).

The results of the study showed that there were differences in the classification of the patient’s general condition according to age and gender on the ASA scale. Spearman’s rho rank correlation coefficient was used to check the former relationship. It turned out to be statistically significant (rs = 0.409; *p* < 0.001), indicating a moderate association between age and the ASA scale score. Statistically, the older the patient, the higher his/her ASA score, and thus the worse the overall condition (Table 4).

The Mann–Whitney U rank test analysis showed that men (M_rank_ = 58.71) achieved significantly higher median ranks on the ASA scale than women (M_rank_ = 46.25), U = 958.500; *p* = 0.016. This indicates a statistically significant difference in the general condition of patients of different sexes qualified for hip arthroplasty surgery at the hospital on Lindleya Street—women in this population are generally healthier (Table 5 and Table 6).

**Hypothesis** **1:** *Anxiety understood as a trait correlates with patient classification on the ASA scale and the Lee index*.

Spearman’s rho rank-order correlation coefficient was found to be statistically significant for both the ASA classification and the severity of anxiety in the patients (Table 7). There is a statistically significant correlation of moderate strength between the ASA scale and trait anxiety (rs = 0.359; *p* < 0.001). The higher the anxiety, the higher the degree on the scale. No such relationship was shown by correlating the Lee index and anxiety severity (trait anxiety STAI, rs = 0.149; *p* = 0.134) (Table 8).

**Hypothesis** **2:** *Personality traits according to the Big Five model correlate with scales used for preoperative patient assessment*.

There was a statistically significant, weak association between the severity of neuroticism and the ASA scale (rs = 0.264; *p* < 0.001). Higher severity of this trait is associated with a higher category on the ASA scale. For the other personality traits from the Big Five model (extraversion, openness to experience, agreeableness, conscientiousness), correlations with the ASA classification were found to be statistically insignificant (Table 7).

For the Lee index, the only controlled variables with which there is a statistically significant relationship are the patient’s age and the severity of neuroticism. For age, Spearman’s rho (rs) is 0.275; *p* = 0.005. The older the patient, the higher the Lee index. In the study group, there was a statistically significant weak correlation between the severity of neuroticism and the Lee index (rs = 0.202; *p* = 0.044). The more neurotic the patients, the higher they are rated according to the Lee index. The other variables controlled in the study showed no statistically significant association with the Revised Cardiac Risk Index (Table 8).

A hierarchical regression model was created to test whether personality traits could influence a patient’s preoperative ASA score. The predictors were trait anxiety and neuroticism. Age and gender of the subjects were also controlled. Other personality traits studied (extraversion, openness to experience, agreeableness, conscientiousness) were excluded from the model based on theory and the lack of statistically significant correlations. The regression model explained more than 34% of the variance and was a good fit to the data (Table 9). Controlled variables—age and gender—explained about 23% of the variance. Personality indicators additionally explained slightly more than 11% of the variance, which was considerable and statistically significant. Anxiety understood as a trait in Spielberger’s concept (Beta = 0.293) proved to be a better predictor than neuroticism from the Big Five model (Beta = 0.054).

## 4. Discussion

The aim of this study was to investigate whether personality traits and anxiety are related to the anesthesiologist’s preoperative assessment on somatic health evaluated in the ASA scale and the Lee index. Considering the assumptions mentioned above, the study analyzed the occurrence of correlations between personality traits (the Big Five model) and anxiety (trait anxiety) with anesthesia scales for preoperative evaluation in patients enrolled for hip replacement surgery.

The results of the study showed that patients with a high severity of trait anxiety and neuroticism had statistically significant higher classification on the ASA scale. The severity of neuroticism, but not trait anxiety, correlated also with the Lee index (the higher the neuroticism, the higher the Lee index).

To derive a robust and comprehensive estimate of the overall relation between Big Five personality traits and health variables, Strickhouser et al. used metasynthesis. They stated that personality predicts overall health and well-being [13]. Denovan et al. found a positive correlation between neuroticism and somatic complaints. They concluded that it could have implications for healthcare in terms of managing individuals who present with multiplate somatic complaints [24]. Moreover, according to Nilsson et al., both mental and physical preoperative status have an impact on patients’ postoperative recovery and a serious attempt must be made as a part of the routine preoperative assessment to include not only the physical but also the mental preoperative state of the patient [25].

The results of our study show that only neuroticism (but not other personality traits) had a correlation with cardiac health measured by the Lee index and general health measured by the ASA scale.

The preoperative ASA scale used worldwide as a somatic health assessment classification was found to be significantly associated with self—estimated anxiety prior to orthopedic surgery in Sweden [26]. Such an increased state of anxiety (provoked by a situation such as surgery) is correlated with higher levels of trait anxiety experience [27].

Our study results showed an association between higher category on the ASA scale and higher severity of anxiety trait. Considering the assumptions mentioned above, more frequent anxiety states can be expected with a higher ASA scale score in perioperative care. Perhaps the introduction of anxiety trait screening would make it possible to introduce a more individualized approach to patients, which could result in better surgical outcomes. Similar conclusions have already been drawn in patients with vestibular schwannoma.

Preoperative psychological examination might help to select the population of patients at risk of postoperative headache after microsurgery and give them an opportunity to choose psychological support in order to optimize treatment [28].

Our study results also showed a moderate association between age and gender and the ASA scale score. Statistically, the older the patient, the worse the overall condition. In the studied population, women generally received lower scores on the ASA scale compared to men. The above patient features account for about 23% of the variance in the ASA classification score (without taking into account the patient’s history of somatic diseases). In addition, trait anxiety and neuroticism explain a considerable (11%) and statistically significant portion of the ASA classification variance. Trait anxiety was found to be a better predictor of ASA scores than neuroticism. High neuroticism, and the high anxiety level associated with it, might moderate the magnitude of organism’s stress responses. They may be correlated with more prolonged sympathetic responses to stressors and greater cardiovascular reactivity [29]. Moreover, neuroticism was found to be associated with increased cardiovascular disease risk and smaller decline in cortisol levels throughout the day, suggesting a sustained Hypothalamic–Pituitary–Adrenal axis activation [30,31]. Other personality traits mainly affect health indirectly, by the individual’s behavioral patterns and lifestyle. On the basis of that theoretical ground, the metasynthesis data created by Strickhouser et al., and the lack of statistically significant correlations found in this study, other personality traits studied (extraversion, openness to experience, agreeableness, conscientiousness) were excluded from the hierarchical regression model [13].

This results of our study may serve as an introduction to further research on the impact of the severity of a patient’s disposition to react to different situations with anxiety on the entire perioperative process—from the enrollment for hip replacement to the patient’s physical therapy after surgery.

## 5. Limitations

Linking personality traits to preoperative patient assessment is a complex task. The analyses carried out do not exhaust the topic, but they provide a theoretical basis for expanding research into the problem. Above all, it is worth expanding the study population. Only 102 patients from one department were included in the above study. A multi-center study with a larger number of patients of different ages may be a valuable broadening of future analyses. This is especially important in the context of the Big Five model personality traits, since the reliability of the NEO-FFI inventory decreases in older people [9].

Future work may also explore another way of operationalizing personality. Despite the dominance of the Five Factor Theory of Personality, it is not without flaws. The NEO-FFI consists of as many as 60 questions and the respondents’ answers may have been influenced by their increasing fatigue as it was being completed.

A single sheet from the STAI psychological questionnaire was used to measure trait anxiety. Another possible way of assessing anxiety could be to measure the severity of symptoms of the generalized anxiety syndrome, for example, through the use of the GAD-7 screening questionnaire. It is likely that the results obtained would lend themselves to more direct medical interpretation and provide greater opportunity for intervention [32].

Improving ways of preoperative patient assessment is also a possible course for future research. The ASA scale is a subjective method and depends on the anesthesiologist who performs it [33]. To reduce the impact of subjectivity, all the patients were evaluated by physicians working in a single clinic (the First Department of Anesthesiology and Intensive Care at the Medical University of Warsaw), but this is undoubtedly insufficient. It would be worthwhile to achieve a greater level of standardization of assessment, for example, by introducing a more objective indicator of the patient’s general condition. Perhaps the mental state and personality traits of patients should also be considered.

## 6. Conclusions

1. Patients with a high severity of trait anxiety are classified higher on the ASA scale;
2. Neuroticism, defined according to the Big Five model, significantly correlates with scales of preoperative patient assessment (the ASA classification and the Lee index);
3. We suggest that personality traits might be included in the preoperative evaluation and personality questionnaires should be included in the patient’s preoperative assessment documentation.

The findings seem to indicate that a significant degree of improvement in patient preparation for surgery can be achieved by involving a clinical psychologist throughout the process. Perhaps screening a patient’s personality on admission to the surgical ward could help identify a group of patients who would particularly benefit from preoperative psychological prophylaxis aimed at reducing perioperative stress.

## Figures and Tables

**Table 1:** ASA scale.

ASA I	A generally healthy patient
ASA II	A patient with a mild systemic disease that does not affect normal functioning
ASA III	A patient with a severe systemic disease limiting normal functioning
ASA IV	A patient with severe systemic disease that is a constant threat to life
ASA V	A patient who is dying and will not survive without surgery
ASA VI	A declared brain-dead patient whose organs are being removed for transplantation

ASA—American Society of Anesthesiology.

**Table 2:** Revised Cardiac Risk Index (Lee index).

1. ischemic heart disease	Total RCRI score and corresponding risk of myocardial infarction, sudden cardiac arrest, or death within 30 days of non-cardiac surgery: 0 predictors = 3.9% 1 predictor = 6.0% 2 predictors = 10.1% ≥3 predictors = 15%
2. congestive heart failure	
3. cerebrovascular disease (stroke or transient ischemic attack)	
4. diabetes mellitus requiring preoperative insulin use	
5. chronic kidney disease (creatinine >2 mg/dL)	
6. surgical procedure with moderate or high risk	

**Table 3:** Quantitative variables analyses.

	Kolmogorov–Smirnov ^a^		
	Statistics	df	Significance
ASA	0.350	102	<0.001
Lee	0.442	102	<0.001
Age	0.149	102	<0.001
stenN	0.165	100	<0.001
stenE	0.101	100	0.013
stenO	0.125	100	<0.001
stenAg	0.133	100	<0.001
stenC	0.163	100	<0.001
stenAn	0.220	102	<0.001

^a^ With the Lilliefors significance correction. Trait anxiety (An), neuroticism (N), extraversion (E), openness to experience (O), agreeableness (Ag), and conscientiousness (C).

**Table 4:** Correlation of the ASA scale and age.

			Age
Spearman’s rho	ASA	Correlation coefficient	0.409 **
		Significance (bilateral)	<0.001
		N	102

** Significant correlation at the level of 0.01 (bilateral).

**Table 5:** Descriptive statistics of ASA scores by gender.

	M/F	N	Average Rating	Total Rating
ASA	FEMALE	59	46.25	2728.50
	MALE	43	58.71	2524.50
	Total	102		

Clustering variable: M/F. M—male; F—female.

**Table 6:** Statistical comparison of ASA scores between women and men.

	ASA
Mann–Whitney	9580.500
W Wilcoxon	27,280.500
Z	−20.416
Asymptotic significance (bilateral)	0.016

**Table 7:** Correlations between the preoperative assessment on the ASA scale and personality traits.

			stenAn	stenN	stenE	stenO	stenAg	stenC
Spearman’s rho	ASA	Correlation coefficient	0.359 **	0.264 **	−0.122	−0.151	−0.182	−0.030
		Significance (bilateral)	<0.001	0.008	0.225	0.133	0.071	0.764
		N	102	100	100	100	100	100

** Correlation is significant at the 0.01 level (bilaterally). Trait anxiety (An), neuroticism (N), extraversion (E), openness to experience (O), agreeableness (Ag), and conscientiousness (C).

**Table 8:** Correlations between the Lee index, the ASA scale, and personality traits.

			ASA	Age	stenAn	stenN	stenE	stenO	stenAg	stenC
Spearman’s rho	Lee	Correlation coefficient	0.475 **	0.275 **	0.149	0.202 *	−0.023	−0.077	−0.035	−0.059
		Significance (bilateral)	<0.001	0.005	0.134	0.044	0.824	0.449	0.729	0.558
		N	102	102	102	100	100	100	100	100

** Correlation is significant at the 0.01 level (bilaterally). * Correlation is significant at the 0.05 level (bilaterally). Trait anxiety (An), neuroticism (N), extraversion (E), openness to experience (O), agreeableness (Ag), and conscientiousness (C).

**Table 9:** Regression analysis of preoperative ASA patient score.

	Model 1	Model 2	95% Confidence Interval for B	
	B	B	Lower Limit	Upper Limit
(Constant)	0.692 *	0.265	−0.360	0.890
Age	0.019 ***	0.019 ***	0.012	0.027
Gender	0.306 **	0.280 **	0.090	0.470
Sten of trait anxiety		0.077 *	0.015	0.138
Sten of neuroticism		0.014	−0.047	0.076
R^2^	0.233	0.343		
F	14.696 ***	12.417 ***		
∆R^2^		0.111		
∆F		8.014 ***		

*** Correlation is significant at the <0.001 level (bilaterally). ** Correlation is significant at the 0.01 level (bilaterally). * Correlation is significant at the 0.05 level (bilaterally).

## Data Availability

The original contributions presented in this study are included in the article. Further inquiries can be directed to the corresponding author.
